# Supplementary material for: Urine Metabolomic Profiling and Machine Learning in Autism Spectrum Disorder Diagnosis: Toward Precision Treatment
Source: Metabolites. 2025 May 16;15(5):332. doi: 10.3390/metabo15050332 (PMC12113876; doi:10.3390/metabo15050332)
Supplement: Supplementary file 1 [file metabolites-15-00332-s001.zip › metabolites-3602969-supplementary.pdf]

Supplemental materials

Table S1: Classification results for all 293 metabolites

Classifier	***Random Forest***	***J48 Pruned Tree***	***Logistic Regression***	***Naïve Base***
AUC	0.9	0.85	0.61	0.61
Accuracy	85%	87%	62%	52%

Table S2: Classification results for 189 Endogenous metabolites.

Classifier	***Random Forest***	***J48 Pruned Tree***	***Logistic Regression***	***Naïve Base***
AUC	0.86	0.85	0.71	0.72
Accuracy	81%	83%	69%	54%

Table S3: Classification results for 104 Exogenous metabolites.

Classifier	***Random Forest***	***J48 Pruned Tree***	***Logistic Regression***	***Naïve Base***
AUC	0.72	0.85	0.47	0.52
Accuracy	71%	83%	60%	60%
